# Structure property relationships and symmetry-mode analysis of single-layered hybrid organic-inorganic perovskite compounds

**DOI:** 10.1063/4.0000967

**Published:** 2025-10-27

**Authors:** Isaac R. Burkholder, Cindy Y. Wong, Kameron R. Hansen, John S. Colton, Andre Schleife, Harold T. Stokes, Branton J. Campbell

**Affiliations:** 1 Brigham Young University, Provo, Utah, USA; 2 University of Illinois Urbana-Champaign, Urbana, Illinois, USA; 3 University of Utah, Salt Lake City, Utah, USA

## Abstract

The low-symmetry crystal structure of an inorganic compound can often be viewed as possessing pseudo-symmetries relating it to a higher- symmetry parent structure, such that the actual structure can be viewed as a distortion of the parent. One can then employ group representation theory to decompose the distortion into symmetry modes belonging to irreducible representations (irreps) of the parent. The physical properties of single-layered hybrid-organic-inorganic perovskite (HOIP) materials are reported to correlate with complex distortions of their inorganic frameworks [1]. Here, we report on observed correlations between the amplitudes of specific displacive symmetry modes of the inorganic framework and features of the DFT electronic band structure across a variety of single-layered HOIP compounds.

